# Electrophoretic Deposition of Copper(II)–Chitosan Complexes for Antibacterial Coatings

**DOI:** 10.3390/ijms21072637

**Published:** 2020-04-10

**Authors:** Muhammad Asim Akhtar, Kanwal Ilyas, Ivo Dlouhý, Filip Siska, Aldo R. Boccaccini

**Affiliations:** 1 Department of Materials Science and Engineering, Institute of Biomaterials, University of Erlangen-Nuremberg, Cauerstr.6, 91058 Erlangen, Germany; asim.akhtar@fau.de (M.A.A.); kanwal.ilyas1@gmail.com (K.I.); 2 Institute of Physics of Materials ASCR, CEITEC IPM, Žižkova 22, 61662 Brno, Czech Republic; idlouhy@ipm.cz (I.D.); siska@ipm.cz (F.S.)

**Keywords:** chitosan, complexes, copper, electrophoretic deposition, antibacterial, coatings

## Abstract

Bacterial infection associated with medical implants is a major threat to healthcare. This work reports the fabrication of Copper(II)–Chitosan (Cu(II)–CS) complex coatings deposited by electrophoretic deposition (EPD) as potential antibacterial candidate to combat microorganisms to reduce implant related infections. The successful deposition of Cu(II)–CS complex coatings on stainless steel was confirmed by physicochemical characterizations. Morphological and elemental analyses by scanning electron microscopy (SEM) and energy-dispersive X-ray (EDX) spectroscopy verified the uniform distribution of copper in the Chitosan (CS) matrix. Moreover, homogeneous coatings without precipitation of metallic copper were confirmed by X-ray diffraction (XRD) spectroscopy and SEM micrographs. Controlled swelling behavior depicted the chelation of copper with polysaccharide chains that is key to the stability of Cu(II)–CS coatings. All investigated systems exhibited stable degradation rate in phosphate buffered saline (PBS)–lysozyme solution within seven days of incubation. The coatings presented higher mechanical properties with the increase in Cu(II) concentration. The crack-free coatings showed mildly hydrophobic behavior. Antibacterial assays were performed using both Gram-positive and Gram-negative bacteria. Outstanding antibacterial properties of the coatings were confirmed. After 24 h of incubation, cell studies of coatings confirms that up to a certain threshold concentration of Cu(II) were not cytotoxic to human osteoblast-like cells. Overall, our results show that uniform and homogeneous Cu(II)–CS coatings with good antibacterial and enhanced mechanical stability could be successfully deposited by EPD. Such antibiotic-free antibacterial coatings are potential candidates for biomedical implants.

## 1. Introduction

Most problems associated with biomedical implants are due to infectious diseases of bacterial origin. Therefore, a current goal of biomaterial technology is to develop antibacterial surfaces for implants that can effectively combat micro-organisms and biofilm formation [1,2,3]. The risk of infections can be reduced by the local delivery of biologically active agents in a controlled manner [4].

In this context, an emerging threat to humanity that has been recently recognized by scientists all over the world is the increasing bacterial resistance to antimicrobial agents, especially antibiotics [5]. Therefore, a strong multidisciplinary counterattack from the scientific community is required, which should involve the development of alternative (antibiotic-free) antimicrobial technologies to control unwanted resistance to bacteria [6]. The need of the hour is to develop advance biomaterials for medical devices, such as modified implant coatings that not only provide an efficient therapeutic solution for patients but also exhibit antimicrobial properties preferable without using antibiotics that can obstruct the attachment and growth of different kind of harmful microorganisms on the surface of implants [7,8,9].

Recently, a material that has gained immense attention for the development of antibacterial coatings is Chitosan (CS) [10]. The main reasons are the excellent beneficial properties of CS, particularly its intrinsic cytocompatibility, antibacterial, and antifungal properties [11]. Moreover, CS possesses several other attractive properties that make it a distinctive candidate for the development of an extensive range of biomedical devices. Among these unique set of characteristics, the most beneficial property of CS is its capability of chelation with a broad spectrum of metal ions, especially transition elements [12]. The chelating ability of CS is well-documented and has been extensively studied [12,13,14]. The strategy involves incorporation of the metallic ions within the polysaccharide matrix, which bring an extra functionality provided by the biological effect of the added metallic ion. Above all, such CS–metal ion complexes are reported to have superior in vitro antibacterial activities as compared to free CS or antimicrobial metal salts [15,16]. Another key advantage of chelation is the ability to release the metal ions from the polymer matrix in a controlled manner [17,18]. Moreover, when choosing the appropriate therapeutic metal ions (TMIs), the chelated complex could assist vital biological processes that include osteogenesis and angiogenesis. TMIs have the ability to interact with several biological structures and metabolic systems and can induce positive effects on the tissue regeneration mechanisms, inhibiting the growth of prokaryotes while interacting with target mammalian cells [19]. In the vast range of transition metal ions, copper is considered one of the best candidates because of its superior antibacterial activity against a huge variety of human pathogens [20] and its potential angiogenic effect [21].

Electrophoretic deposition (EPD) is a widely adopted method for the deposition of biopolymers and organic and inorganic coatings on metallic substrates due to its effectiveness and versatility [22]. During EPD, charged particles or molecules move in a liquid medium and deposit on the electrode (e.g., a metallic substrate) under the application of an electric field [23]. Being economical and a room-temperature process, EPD is one of the most widely used techniques for producing thin and thick coatings, the coating thickness and homogeneity can be easily controlled through simple adjustment of EPD parameters [23]. According to the literature, there have been few attempts to co-deposit CS with various TMIs by EPD, using a simultaneous cathodic deposition. However, the results from such previous studies indicate that atomic deposition of the metallic phase and the formation of new crystalline phases occur on the electrode after EPD [10,24,25,26,27]. In such cases metal particles physically embedded in the chitosan matrix and furthermore the addition of salts in the deposition suspension significantly effects CS electrodeposition behavior, which leads to the formation of less homogeneous and less rigid films [28,29]. 

Therefore, the emphasis in the present research approach has been to develop CS-based coatings on metallic substrates by EPD exploiting the chelation between CS and Cu metal ion prior to EPD. In order to take advantage of the properties of CS–metal complex material, as well as to produce homogenous coatings without the formation of additional metal species, a new alternative to the single-step deposition is required. In this work, a new two-step EPD coating procedure is proposed, which consists of the synthesis of Copper(II)–Chitosan (Cu(II)–CS) complexes followed by the EPD of the synthesized Cu(II)–CS complex on the metallic substrate. The success of this method is due to the fact that the soluble polymer–metal ion complex behaves as a polyelectrolyte in aqueous solutions, so that a continuous and crack-free coating with improved antibacterial efficacy could be prepared. To the best of the authors’ knowledge, the current research is the first effort at combining CS–Cu(II) complexes to obtain robust homogeneous coatings by EPD with potential biomedical application.

## 2. Results and Discussion

### 2.1. EPD Process of Cu(II)–CS

It is well known that CS is a powerful chelating agent, which easily forms complexes with transition metals and heavy metals. Chelation is the formation of multiple coordination bonds between organic molecules and a transition metal ion. For the purpose of EPD we dissolved the Cu(II)–CS complex prepared by the method described above, in an ethanol–acetic acid solution at pH 4.7. It is well known that at this pH, amine groups of CS get protonated; however, CS complexes are stable and they maintain their chemical integrity at this pH [30]. Moreover this pH is favorable for the adsorption of metal ions in CS [30,31,32,33]. However, in highly acidic solutions the adsorption capacity of CS for cations is lowered because the higher H^+^ ion concentration reduces the number of binding sites for metallic ions [34]. Moreover, the Cu(II) ion is connected with the amine and OH groups of CS forming a bridge via coordinate covalent bond that is strong enough to maintain the complex structure [30]. Under the effect of the applied voltage for EPD, this complex, which is composed of positively charged CS molecules and the metal cation Cu(II) move toward the negative electrode. Due to the electrochemical decomposition of water, the local pH around the cathode surface increases, which helps to deposit Cu(II)–CS as a film on the surface of the substrate following the deprotonation of the CS. 

Table 1 shows the zeta potential of suspensions at a pH of 4.7. As expected, the positive values predict a cathodic deposition under the application of an electric field. In the case of Cu(II) complexes, it was determined that with the increase in copper concentration zeta potential is reduced because copper forms polydentate with the amine groups of CS. With the increase in copper concentration, more amine groups are involved in the complexation, which causes the depletion of protonated sites on CS. However, it was observed that at higher concentrations of Cu(II), the zeta potential remains constant, which is likely because chelation of Cu(II) with amine group is established inside the 3D structure formed by the polysaccharide chains. 

### 2.2. Morphological Analysis

**Figure 1:**
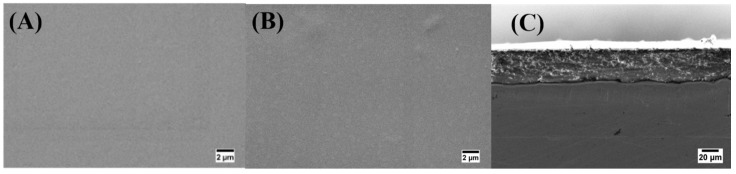
Scanning electron microscopy (SEM) morphologies of coatings (**A**) Chitosan (CS), (**B**) Copper(II)–Chitosan (Cu(II)–CS), and (**C**) cross-sectional image of Cu(II)–CS4, indicating a coating thickness of around 40μm.

The scanning electron microscopy (SEM) study elucidated the morphology of EPD coatings, the micrographs are presented in Figure 1. Here, uniform CS and Cu(II)–CS coatings can be seen in both top and cross-sectional views of the coatings. However, even at higher magnification the morphology of the Cu(II)–CS4 and CS coatings revealed no significant difference. It is evident from SEM micrographs that Cu(II)–CS coatings did not show the formation of the second phases or existence of copper particles, which confirms the successful homogeneous deposition of the CS(II)-CS complexes. Besides that, the addition of Cu(II) does not seem to lead to any significant qualitative difference in the structure of the coatings. These results confirm the deposition of homogeneous Cu(II)–CS layer by EPD, a result reported here for the first time. Furthermore, the thickness of all coatings was measured using SEM micrographs, which revealed a thickness of 40μm ± 1μm along with a well adherent appearance of the coatings to the substrates. Therefore, the results strongly support the evidence that the optimized EPD parameters are suitable for homogeneous and crack-free coatings. Moreover, the coatings were shown to be firmly bonded to the substrate without underneath cracks.

### 2.3. Chemical and Structural Characterization

**Figure 2:**
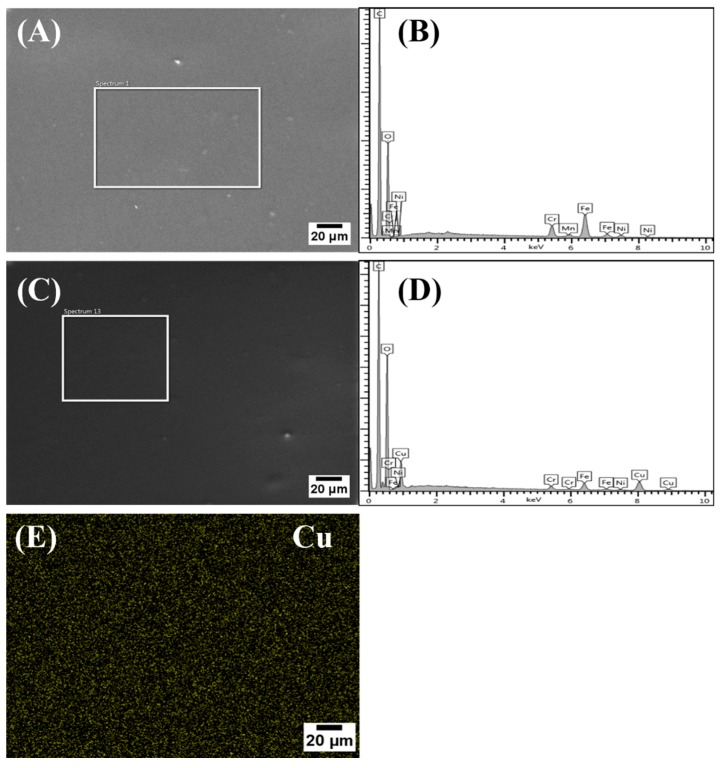
EDX spectra of coatings: (**A**) SEM image of CS, (**B**) EDX pattern of CS, (**C**) SEM image of Cu(II)–CS4, (**D**) EDX pattern of Cu(II)–CS4, and (**E**) Cu mapping on Cu(II)–CS4 coating.

The elemental compositions of the deposited coatings were investigated by energy-dispersive X-ray (EDX) analysis. EDX spectra of the CS and Cu(II)–CS4 are shown in Figure 2. It is evident in the EDX spectra that CS coatings displayed the discernible peaks of carbon and oxygen that are key elements of CS. Moreover, the peaks of various other elements (Fe, Mn, Cr, Ni) in the EDX spectra correspond to the stainless-steel substrate. The existence of Cu in the coating can be seen in the spectra (D) of Cu(II)–CS. This was also detected in the coatings of other synthesized Cu(II)–CS complexes (not shown here). Additionally, the homogeneity of copper in the coatings is confirmed by the mappings across the sample. Notably, an important finding is that EDX results did not reveal any contamination in the synthesis of the Cu(II)–CS powder and the EPD coatings. 

In a previously reported method [16], a semi-quantitative evaluation of Cu concentration in the complexes was done. In the same way, evaluation of Cu concentration in the present coating was conducted. From various spectra (*n* = 3), the area of the Cu L peak was normalized with respect to the carbon peak. It was found that Cu_kL_/ C_kα_ ratio increased proportionally to the amount of Cu added to the CS (Table 2), which confirms that the composition of the coatings was consistent with the as-prepared Cu(II)–CS complexes and was also in agreement with the theoretical values. This result also implies that complexes retain their chemical composition during EPD. 

**Figure 3:**
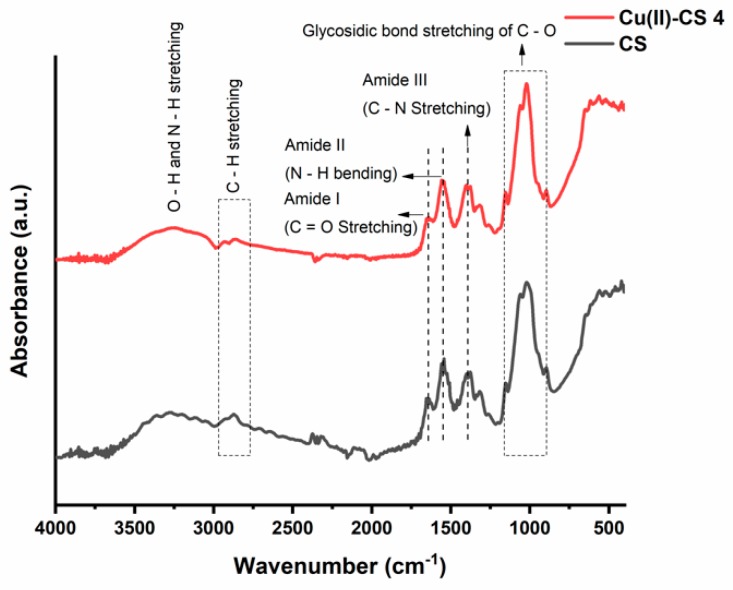
Fourier-transform infrared (FTIR) spectra of CS and Cu(II)–CS4 coatings. The relevent peaks are discussed in the text.

Functional groups were determined with the help of Fourier-transform infrared (FTIR) spectroscopy. The FTIR spectra of prepared coatings are shown in Figure 3. A graphical comparison is made in which a CS coating is discussed as a reference and studied with respect to the Cu(II)–CS4 coating. The findings show no quantifiable differences in the spectra of all samples with different amounts of Cu. FTIR spectra of CS and Cu(II)–CS4 consist of a broad band at around 3250 cm^−1^ that is attributed to the overlapping of N–H, O–H bond stretching and hydroxyl groups from the adsorbed water [35,36]. The absorption band located at 2860 cm^−1^ is ascribed to the stretching of C–H bond [36]. Whereas, the peaks of amide I at 1650 cm^−1^ and amide II at 1550 cm^−1^ are due to the stretching of C=O and bending of N–H bonds [37]. The absorption peak around 1400 cm^−1^ is either due to the deformation of the C–H or the stretching of C–N bond [16,30,36]. The band with various small peaks in the region of 900 cm^−1^ to 1160 cm^−1^ is attributed to the glycosidic bond stretching C–O–C that connects the glucosamine monomers of CS [16,37]. 

The structural changes in CS occur due to the chelation of CS with Cu(II) ions. This modification causes the change in relative absorbance of specific spectral bands that participate in the complex formation of Cu(II) and CS. A decrease in intensities at 1650 cm^−1^ and 1550 cm^−1^ is also observed, that appears predominantly because of the C=O and N–H stretching vibrations. The literature related to complex formation reports similar observations, confirming that these groups are involved in the formation reaction [16,36]. Moreover, the change in shape of the C–O characteristic peak at 1020 cm^−1^ was also observed, which is possibly due to the increase in length of glycosidic bond by steric effects following Cu(II) cross-coordination with adjacent chains of CS [16]. This result confirms that, during EPD, Cu(II)–CS complex coatings were obtained.

**Figure 4:**
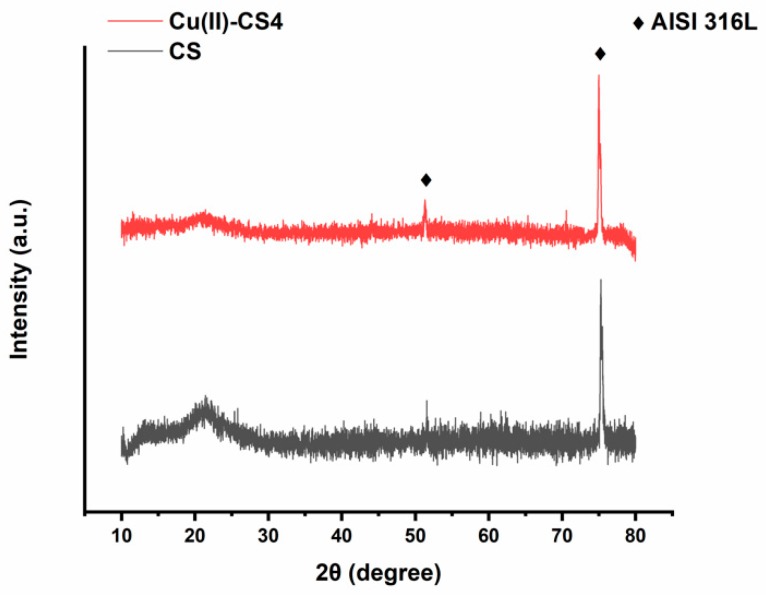
X-ray diffraction (XRD) patterns of CS and Cu(II)–CS4 coatings.

The crystallographic patterns of pure CS and Cu(II)–CS coatings are shown in Figure 4. The XRD pattern of CS coating shows two characteristic broad diffraction peaks at 2θ = 12^o^ which reveals the amorphous nature, whereas the peak at 2θ = 20^o^ indicates a high degree of crystallinity in the CS structure [18,38]. However, the XRD pattern shows also two more peaks at 2θ = 51^o^ and 75^o^ indicating the presence of the stainless-steel substrate. The sample having Cu(II)–CS4 coating shows a slightly different pattern, the characteristic peaks of CS are weakened and have almost disappeared. The fact behind this change in crystallinity is that the complexation of CS with Cu(II), which caused a reduction of available binding sites for hydrogen bonding (NH_2_ and OH), results in decreasingnumber of inter- and intramolecular bonds between CS chains necessary for self-assembly of the polysaccharide. However, no significant differences were observed between coatings with different levels of Cu(II). Moreover, no crystalline peak was observed which would indicate the presence of metallic Cu particles. This confirms that Cu(II) forms a complex and did not precipitate as metallic Cu.

### 2.4. Nano Indentation

**Figure 5:**
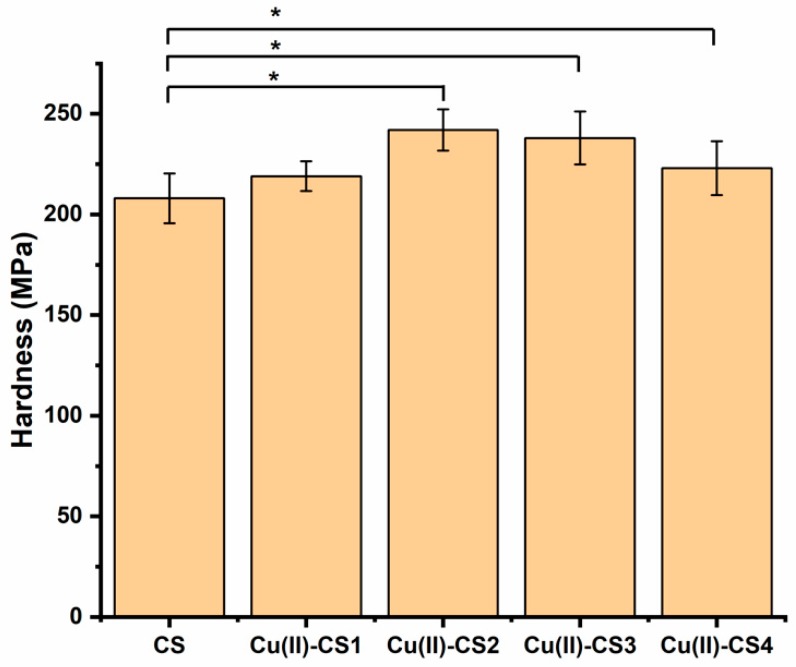
Overall variation of the hardness of the different coatings. Significantly different coatings from the CS coating are highlighted (* *p* < 0.05).

Mechanical properties play a crucial role in the stability and long-term performance of biomaterials. The hardness of a material can be defined as a measure of its resistance to a permanent shape change when a constant compressive force is applied and thus describes mainly the portion of plasticity in the material behavior [39]. The measurement of hardness was conducted on the present coatings because this property is important in relation to the wear resistance. Results from nanoindentation experiments of coatings of CS and complexes of CS with different concentrations of Cu are presented in Figure 5. The hardness of all investigated coatings is in the range of 210–240 MPa. It is apparent that with the addition of Cu(II) ions the hardness of CS coatings gradually increased up to a certain limit (Cu(II)–CS3). The fact is attributed to the effect of Cu on the mobility of the polymer chains, which results in the decrease in plastic deformation and increase in brittleness. This result agrees with previously published studies on CS films, which demonstrated that crosslinking agents can be used to improve hardness [40,41]. However, at a higher concentration of Cu(II), i.e., in case of Cu-CS4, the hardness of the coating decreases. Qu et al. report that the mechanical properties of the complex increase up to certain limit and then start to decrease. The reason for this decrease was considered to be the involvement of a too high concentration of metal ions in complexation, which caused cracks [36]. According to these results, one can suggest that the mechanical properties of CS coatings in terms of hardness can be improved by a properly adjusted concentration of metal ions during synthesis. 

### 2.5. Scratch Test

Scratch test was performed on each type of coating to determine the critical load leading to coating rupture, which is used to compare the cohesive or adhesive properties of coatings. Table 3 shows the critical loads of all coating samples. It was observed that, with an increase in Cu(II) content, the critical load increased, which is due to the complexation and coordination of Cu(II) with CS resulting in increased adhesive properties of the coatings. However, it was also observed that, at the highest concentration (Cu(II)–CS4), the critical load started to decrease.

**Figure 6:**
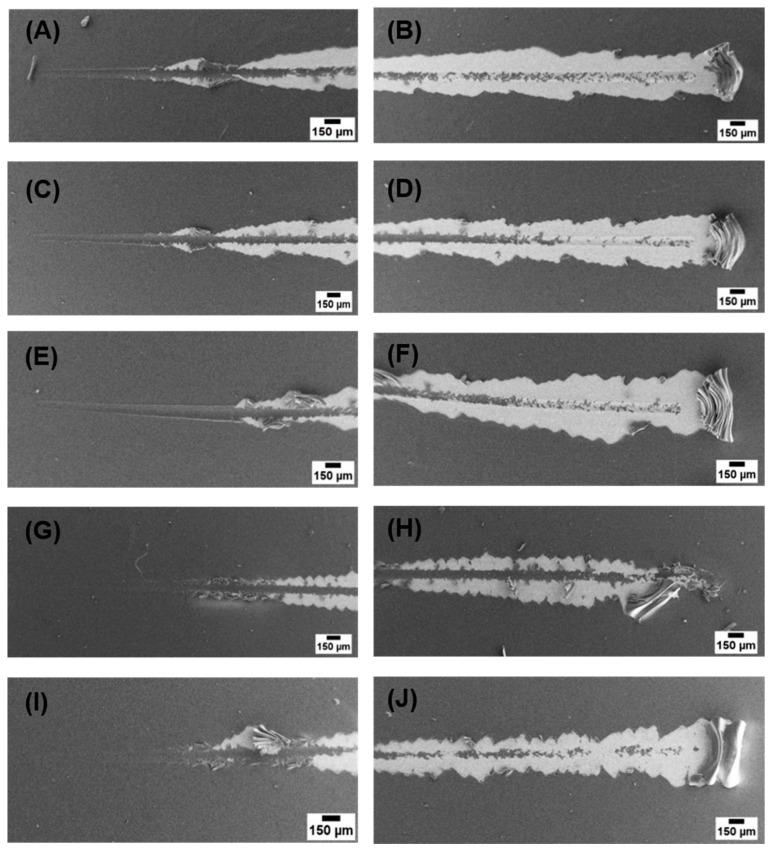
SEM images of sctratchs on coatings at lower and higher loads (**A**,**B**) CS, (**C**,**D**) Cu(II)–CS1, (**E**,**F**) Cu(II)–CS2, (**G**,**H**) Cu(II)–CS3, and (**I**,**J**) Cu(II)–CS4 coatings.

Figure 6 shows micrographs of full scratches on the coatings, from these micrographs it can be seen that CS and the complexes with low concentration of Cu(II) showed plastic deformation at lower loads before delamination. However Cu(II)–CS3 and Cu(II)–CS4 samples showed spallation of coatings rather than plastic deformation, which is the characteristics behavior of brittle coatings [42]. 

### 2.6. Swelling Ratio

**Figure 7:**
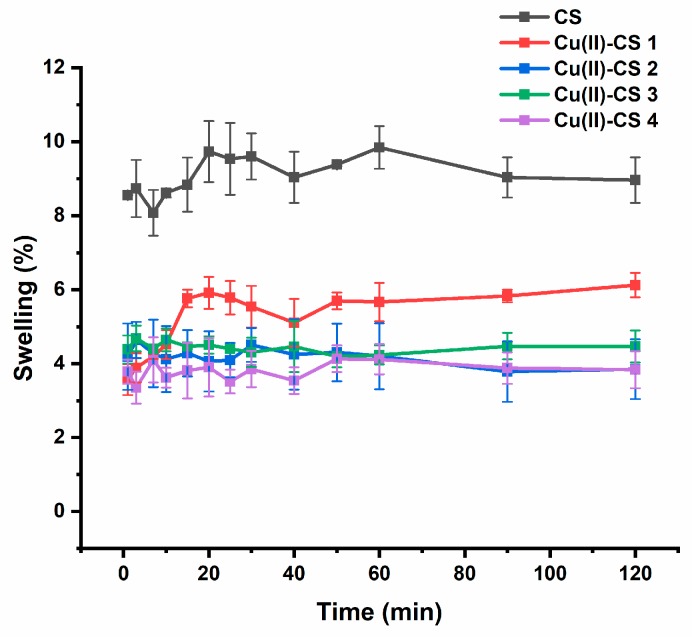
Swelling behaviour of CS and Cu(II)–CS complex coatings with different Cu(II) concentrations with respect to time. Tests were carried out in phosphate buffered saline (PBS).

Swelling capacity defines the ability of a polymer to absorb water [43]. The swelling behavior of Cu(II)–CS coatings was determined at different intervals up to 120 min, as shown in Figure 7. It was observed that CS and Cu(II)–CS1 exhibited a gradual increase in swelling within a certain period of time (around 20 min) and after that the swelling ratio remains approximately constant for the whole course of the study. However, CS coatings showed maximum swelling behavior, which reduced with an increasing amount of Cu(II). In the case of CS coatings, the hydrogen bonding with water and free NH_2_ groups of CS play a main role in the swelling process [44]. The electronegative covalent bonds (N–H) create sites of high polarity that support the rearrangement of water molecules around them [45]. The most probable reason for the relatively low swelling of Cu(II)–CS coatings is the remaining unreacted NH_2_ groups in CS and the chelation of the Cu(II) ion by two adjacent CS chains, which induces a crosslinking of the polysaccharide matrix hindering the polymer chain mobility and restricting water penetration in the network, which causes lower swelling ability. The weight of the coatings after complete drying was checked, and it was found that all coatings regained their initial weight, which indicates the reversible swelling behavior of the present coatings. 

### 2.7. In Vitro Degradation

**Figure 8:**
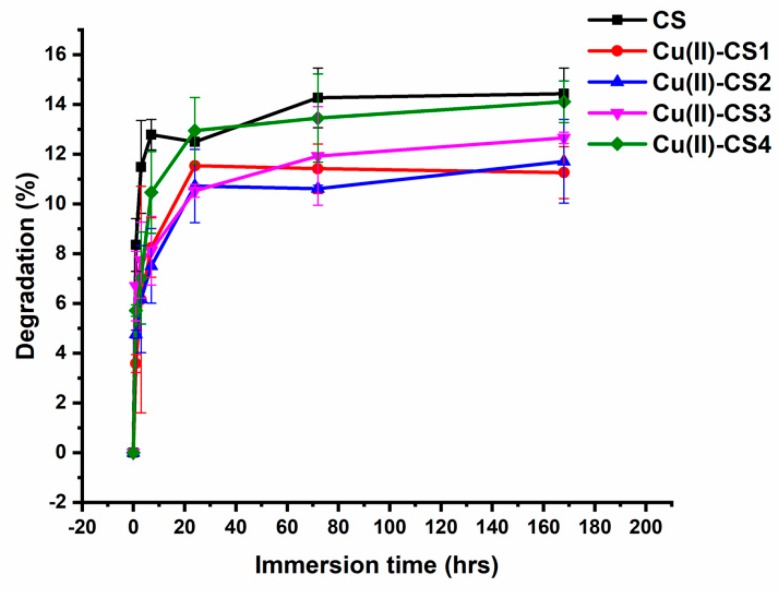
Degradation behaviour of CS and Cu(II)–CS coatings with different Cu(II) concentrations with respect to time in lysozyme–PBS solution.

The weight loss of all coatings was observed as a function of incubation time in a lysozyme– phosphate buffered saline (PBS) solution (pH 7.4) for 1 h, 3 h, 7 h, 3 d, and 7 d. The degradation of all coatings increased sharply within the first 24 h of incubation. No significant variations in degradation were observed for pure CS as well as with Cu(II)–CS coatings having different concentrations of Cu(II). With increasing time, the degradation rate was shown to slows down and finally stabilized after the initial incubation period as shown in Figure 8. The total weight loss detected during the whole degradation period of 1–7 days was in the range of 14.3–14.6%. 

Degradation patterns with high rate of initial mass loss followed by a relatively slower degradation rate were observed for all coatings. This phenomenon can be explained by the mechanism directed by the action of the enzyme. Lysozyme contains a hexameric binding site, a hexasaccharide sequence that presents 3–4 or more acetylated units, which contribute mainly to the initial degradation rate of N-acetylated CS [46]. With increasing time, the degradation progressed at a slower rate, which is the consequence of the loss of appropriate hexasaccharide sequences, leading to decelerated weight loss [47]. The interaction between lysozyme and CS generally includes the following processes: first, the enzyme diffuses from the surrounding solution to the surface of the material, and the subsequent adsorption leads to the formation of enzyme-material complex. Then, the enzyme starts catalysis reactions which involve macromolecule cleavage, causing the release of degradation products into the solution [48,49]. Since the pH of the PBS–lysozyme solution was measured to be 7.4, it can be concluded that in this study no weight loss due to dissolution of CS takes place. The degradation was thus assumed to be dictated only by the mechanism of molecule cleavage because of lysozyme activity.

### 2.8. Wettability

**Figure 9:**
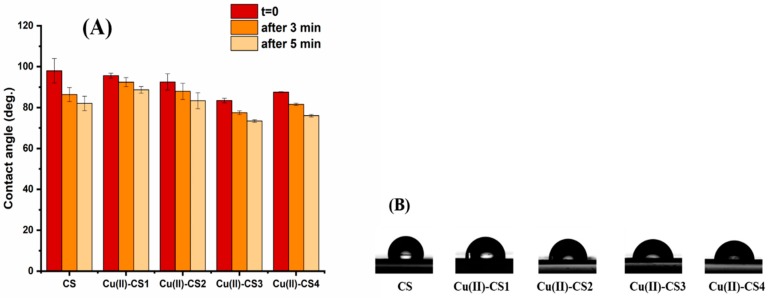
(**A**) Average contact angle of water droplets on CS and Cu(II)–CS coatings measured at three timepoints (i.e., immediately after deposition, after 3 min and 5 min) and (**B**) profiles of water droplets on CS and Cu(II)–CS coatings immediately after deposition.

In order to reveal the degree of wettability contact angle measurements were performed as they give initial information about hydrophilicity of the coating material. Figure 9 shows the contact angle values of coatings with different Cu(II) ion concentrations with respect to deposition time. It is observed that CS and Cu(II)–CS coatings exhibit an initial contact angle in the range of 83–98° regardless the concentrations of Cu(II). Although the behavior of the droplet on the samples appears relatively unchanged after deposition, a different picture can be appreciated by monitoring the change of contact angle over time.

It can be seen that the contact angle on CS coatings reduces significantly with the passage of time. However, the coatings containing different concentrations of Cu(II) did not show a substantial reduction, as compared to CS coatings. The most valid reason is the chelation of the Cu(II) ion with two adjacent CS chains. This chelation induces a crosslinking of the polysaccharide matrix which can reduce cracks on the surface that cause sorption of water from the droplet to the inner part of the coating.

### 2.9. Bacterial Culture

**Figure 10:**
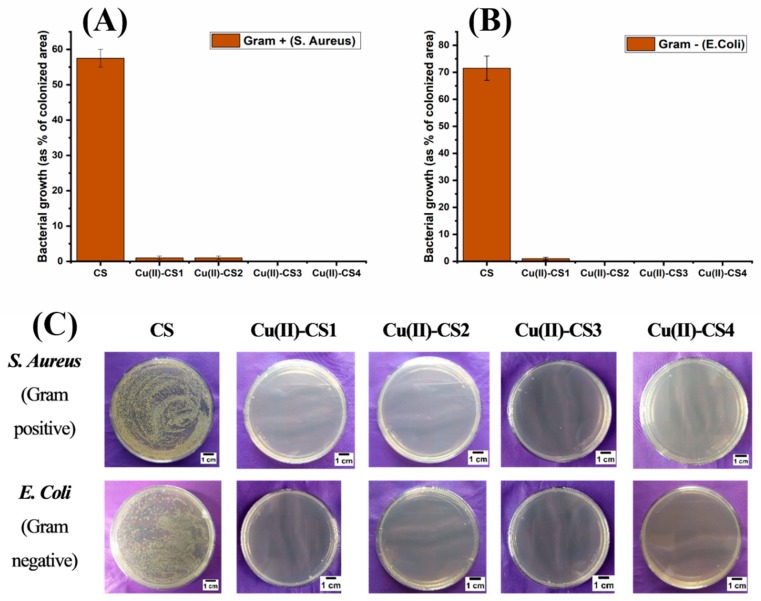
Bacterial growth as % of colonized area: (**A**) *Staphylococcus aureus* and (**B**) *Escherichia Coli*. (**C**) Optical Images of recultivated bacterial colonies on agar after 3 h of incubation for the different samples investigated.

The antibacterial capability of all coatings is shown in Figure 10. It can be seen that all type of coatings with Cu(II) complexation showed a strong antibacterial effect against both Gram-positive and Gram-negative bacteria within the inoculation time of 3 h. The antibacterial effect was also observed for 9 h, 12 h, and 24 h, and there was no significant bacterial growth within this period of time. On the other hand, coating with pure CS did not exhibit antibacterial activity. The antibacterial activity of CS depends upon various factors, including DDA, molecular weight, and environmental conditions such as pH.

It is clear from the previous findings from the release study that even Cu(II)–CS1 releases sufficient amount of Cu(II) ions within 3 h of incubation, which is greater than the minimal inhibitory concentration (MIC, 10–12 ppm). Moreover, Cu(II) was released up to 24 h, and no further release was observed, which is likely due to the chelation of Cu(II) ions [18]. However, further (long-term) release is also possible due to the enzymatic degradation of CS. The additional benefit of this phenomenon exhibited by the present Cu containing CS coatings is that they are capable of inhibiting bacterial growth in the early stages of implantation without causing cytotoxic effects. As an antimicrobial agent, CS–metal complexes have the advantage of showing a higher antibacterial activity than CS and a lower toxicity than Cu [15]. In this way, one can find the suitable concentration range of Cu(II) that is sufficient to kill bacteria without remarkably damaging mammal cells. The present results reveal that Cu(II)–CS coatings effectively inhibit the growth of both chosen strains of prokaryotes (*S. Aureus* and *E. Coli*).

### 2.10. Cell Biology

**Figure 11:**
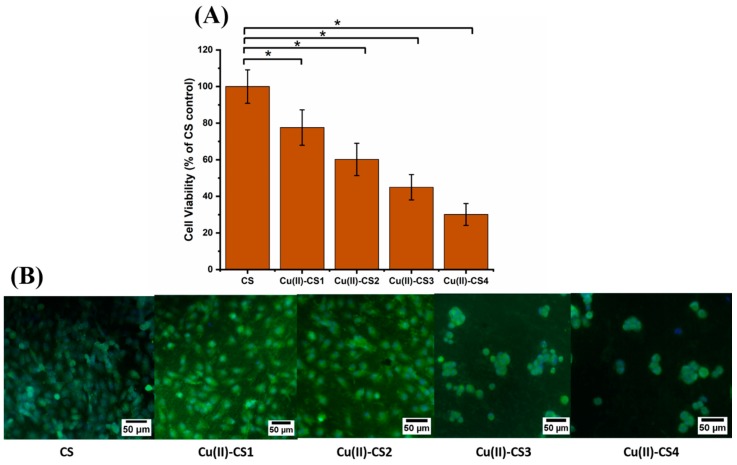
(**A**) Graph representing MG-63 cell viability (WST-8 assay) on different samples investigated, (* *p* < 0.05). (**B**) fluorescence microscope images showing the results of calcein-DAPI staining after 24 h of culture with CS and Cu(II)–CS coatings with different concentration of Cu(II).

The biological characterization was done on each coating by using MG-63 cells in order to assess the cellular activity in the presence of different concentrations of Cu(II) ions. The results showed a statistical reduction of cell viability because of the increase in Cu(II) ion concentration, as shown in Figure 11A. Cu(II)–CS1 and Cu(II)–CS2 coatings depict 78 ± 9 % and 60 ± 8 % cell viability. However, Cu(II)–CS3 and Cu(II)–CS4 reveal a significant decrease in cell viability, i.e., 45 ± 7% and 30 ± 6%, respectively. The assessed cell viability values of these two groups (Cu(II)–CS3 and Cu(II)–CS4) are below 50% of the CS control that indicate cytotoxic effects due to the high concentration of Cu(II) ions. It is worthy to state here that all samples showed lower viability as compared to the previous study on similar Cu(II)–CS complexes [16]. However, the previous study was performed by an indirect method, whereas in the current study, cells were directly seeded on the surface of the coatings. The surface properties of the coatings might play a significant role in the attachment and spreading of the cells that result in the reduction of cell viability. It can be observed that in both studies the samples with lower concentration of Cu(II) ions showed cytocompatibility. Fluorescence microscopy images shown in Figure 11B represent the morphology of cells which confirms the quantitative assessment and reveal how the number of healthy cells tends to decrease with increasing amount of Cu(II) ions. It can be seen that cell morphologies in Cu(II)–CS1 and Cu(II)–CS2 samples are comparable to the CS control. However, on the other two samples, (Cu(II)–CS3 and Cu(II)–CS4), which contain higher concentrations of Cu(II) ion, cells are round in shape. This type of morphology indicates that cells were stained due to the high concentration Cu(II). 

## 3. Experimental Section

### 3.1. Material 

CS, medium molecular weight from Sigma-Aldrich, (Taufkirchen, Germany) (DDA ∼75–85%, MW ∼190–310 kDa, viscosity 200–800 cP), was chosen due to its DDA, which is optimal to load the desired amount of Cu(II) ions. Moreover, the medium molecular weight ensures suitable mechanical strength of chitosan films. Cu (II) chloride dihydrate (purity 99.99%) and lysozyme from chicken egg white were purchased from Sigma Aldrich, Taufkirchen, Germany. Sodium hydroxide (NaOH), ethanol (99%), and PBS tablets were obtained from VWR, Darmstadt, Germany. All reagents were of analytical grade and were used without any further purification.

### 3.2. Synthesis of Cu(II)–CS Complex

Cu(II)–CS complex was synthesized by the previously reported protocol [16]. Briefly, CS incorporating copper was prepared by the in situ precipitation method. CS with the concentration of 2% w/v was dissolved in acetic acid solution (2% *v*/*v*) at 40 °C under constant stirring. After complete dissolution of CS, various amounts of copper salt were added to prepare four different samples with desired ratios of Cu(II) ions to free amino groups, given in Table 4, which was calculated by using the following formulas:

(1) $$m_{CuCl_{2}\cdot 2H_{2}O}=X\times MM_{CuCl_{2}\cdot 2H_{2}O}\times \frac{m_{Cs}}{\bar{MM}}$$

(2) $$\bar{MM}=DDA\times MM_{glu}+\left(1-\mathrm{DDA}\right)\times MM_{N\text{-}\mathrm{acetylglu}}$$

where mCuCl_2_·2H_2_O and MMCuCl_2_·2H_2_O are the mass and molecular mass of the Cu (II) chloride dihydrate, respectively, m_cs_ is the mass of the CS in grams, and MM_glu_ and MM _N-acetylglu_ are the masses of glucosamine (179.17 g/mol) and N-acetylglucosamine (221.21 g/mol) moieties, respectively. The resultant X is the fraction that represents the desired ratio of Cu(II) ions to free amino groups.

After addition of Cu(II) chloride dehydrate, stirring was conducted for one hour so that homogeneous solution was obtained. The solution was added slowly by using pipette in a 0.1 M NaOH solution. The suspension of particles was stirred for another 2 h at room temperature. The obtained particles were filtered and washed with deionized water for complete neutralization of the pH. After complete neutralization, the prepared gels were filtered and dried in an oven at 60 °C overnight. The same protocol was used for the preparation of the CS control without copper.

### 3.3. EPD of Cu(II)–CS

First, 1 g/L Cu(II)–CS particles were dissolved in 20 vol % distilled water and 1 vol % acetic acid by magnetic stirring. After complete dissolution, 79 vol % ethanol was added in that solution to minimize the hydrolysis of water during the EPD process [50]. After that the solution was stirred magnetically for 30 min for homogeneous mixing of Cu(II)–CS and to reduce the air bubbles in the suspension. The stability and charge of the suspension was determined by zeta potential measurements using a zetasizer (nano ZS equipment, Malvern Instruments™, Malvern, UK). Polished stainless steel 316 L foil was used as deposition electrode. The EPD process is versatile enough to be applied to almost all conducting materials, so the results of this study are also applicable to other substrates (e.g., Ti alloys). The EPD of Cu(II)–CS was performed by DC-EPD (Thurlby Thandar Instruments (TTi) EX752M power supply, Huntingdon, UK) at 15V for 10 min at room temperature by keeping the inter-electrode distance at 10 mm. These parameters were optimized by a Taguchi “design of experiment” approach similar to the one previously reported [51].

### 3.4. Characterization of the Coatings

#### 3.4.1. Morphological Analysis

A field emission scanning electron microscope (FESEM, LEO 435VP, Carl Zeiss™ AG, Jena, Germany) was used to analyze the surface and cross-sectional morphology of the coatings. Prior to FESEM analysis, samples were sputter coated (Q150/ S, Quorum Technologies™, Lewes, UK) with gold to avoid the effect of charging on the sample. Furthermore, the thickness of the EPD coatings was estimated by using FESEM cross-sectional imaging. Afterward, ImageJ 1.5i software (National Institutes of Health, USA) was used to calculate the thickness values by averaging the values from 10 different locations on each sample.

#### 3.4.2. Chemical and Structural Characterization

Compositional analysis was performed by using energy-dispersive X-ray spectroscopy (EDX) at 20 kV (LEO 435VP, Carl Zeiss™ AG, Jena, Germany) from different positions of each sample using a Silicon Drift Detector (SDD) X-Max, Oxford Instruments, High Wycombe, UK and Fourier-transform infrared spectroscopy (FTIR; Shimadzu IRAffinity-1S, Shimadzu Corp, Tokyo, Japan) equipped with Labsolution IR software by Shimadzu. Spectra were collected with 40 scans and resolution of 4 cm^−1^ in absorbance mode for wavenumber values ranging from 4000 to 400 cm^−1^. X-ray diffraction (XRD) was performed for structural analysis of CS and CS(II)-CS complex coatings by using X-ray diffractometer (Miniflex 600, Rigaku Corporation, Europe, Neu-Isenburg, Germany) in the 2θ range of 10° to 80° with a step size of 0.010° and dwell time of 1° per minute. Cu Kα radiation was used.

#### 3.4.3. Mechanical Characterizations

Nanoindentation tests were performed at the Institute of Physics of Materials ASCR, Brno, Czech Republic using the Zwick/Roell ZHN Universal Nanomechanical Testing System, Ulm, Germany. A pyramidal diamond indenter was employed for the indentation experiments. All coating samples were mounted on flat aluminum stubs and fixed using super glue. To ensure that the substrate does not interfere with the coating hardness value, different loads from 1 mN to 200 mN were applied on the coatings. It was found that in the range of 1 mN to 5 mN the hardness values were constant, however at higher loads the hardness increased that was surely due to the substrate effect on the measurement. The maximum load of 5 mN was therefore used for this study. The load was held at a maximum value (5 mN) for 60s. Twenty indentations were performed on each sample. Each indent was exactly 100 µm away from the other to avoid the interaction between the plastic strain fields created by each indentation. Hardness values (H) were obtained using Zwick/Roell ZHN software (InspectorX, Ulm, Germany). 

The existence of adhesive or cohesive failures of the coatings was observed by using a CSM Instruments scratch tester (Peuseux, Switzerland). A controlled scratch (*n* = 3) with a Rockwell diamond tip was used with tip radius of 200 μm under linear progressive load from 1 N to 10 N with loading rate of 3.6 N/min and speed of 2 mm/min. The scratch length was 5 mm. In a typical experiment, at some critical load, the coating starts to fail. The point at which the critical loads were achieved was detected using optical photographs. For high resolution imaging of the scratched surfaces, SEM was also performed.

#### 3.4.4. Swelling Ratio

Swelling characteristic of Cu(II)–CS coatings was determined by immersing the coated specimens into 10 mL phosphate buffered saline (PBS) solution for 1, 3, 7, 10, 15, 20, 25, 30, 40, 50, 60, 90, and 120 min. The swelling ratio was determined according to the following formula:

(3) $$\mathrm{Swelling}\;\mathrm{ratio}\;\left(\%\right)=\frac{W_{w}-W_{d}}{W_{d}}\times 100$$

where W_w_ is the weight of the wet specimen immediately after removal from the solution at different time points and W_d_ is the weight of the dry specimen. Three samples of each system were analyzed.

#### 3.4.5. In Vitro Degradation

The degradation study was performed by immersing the coatings for different time intervals (1 h, 3 h, 7 h, 24 h, 3 days, 7days) in phosphate buffered saline (PBS, pH 7.4) at 37 °C containing 1.5 μg mL^−1^ lysozyme. The concentration of lysozyme was chosen to correspond to the concentration in human serum [52,53]. After incubation at predetermined time intervals specimens were removed from the solution and carefully dried overnight at 37 °C. The specimens were weighted to measure the weight loss, which was determined according to the following formula:

(4) $$\mathrm{weight}\;\mathrm{loss}\;\%=\frac{W_{1}-W_{2}}{W_{1}}\times 100$$

where W_1_ is the weight of the dry coating specimen before immersion in lysozyme solution and W_2_ is the weight of the sample after degradation at different time points.

#### 3.4.6. Wettability

Contact-angle measurements were performed at room temperature using a Krüss DSA30 Drop Shape Analysis System (Krüss GmbH, Hamburg, Germany) on dried samples to evaluate the wettability of the coatings. The procedure of this measurements was the deposition of a 3 μL deionized water droplet on the surface of the coatings. Afterwards the computation of the contact-angles from both sides of the droplet (left and right) was done and images of the droplet were acquired by using the software DSA4 (Krüss GmbH, Germany). These measurements were done for a time interval from 0–3 min, and they were carried out five times on different positions on the samples. The goal was to evaluate the changes in contact angle with the passage of time.

#### 3.4.7. Bacterial Culture

*Staphylococcus aureus* (*S. aureus*, ATCC 25923, a gram-positive bacterium) and *Escherichia Coli* (*E. coli*, ATCC25922, a gram-negative bacterium) were used to detect the antibacterial capability of the coatings through in vitro experiments. Both bacteria were cultivated in lysogeny broth (LB) medium and on Luria-Bertani (LB) agar plates at 37 °C. In order to evaluate the coating’s ability to inhibit bacterial growth over time a direct contact bacterial assay was performed on all types of coatings. All glassware was sterilized in autoclave and the samples were sterilized by UV irradiation for 1 h. Isolated colonies of Gram-negative and Gram-positive bacteria selected as test strains were cultured in a nutrient broth (LB) overnight at 100 rpm and 37 °C in incubator. On the second day, the fresh bacteria suspension was diluted to an optical density of 0.015 (600 nm, Thermo Scientific™ GENESYS 30™, Schwerte, Germany). Thirty microliters of diluted bacterial suspension (∼1 ×10^7^ colony forming units (CFU)/mL) were dropped onto the sample surface and cultured for 3 h, 6 h, 9 h, and 24 h at 37 °C. For each time point, different coatings of each system were used. For the significance of the experiment, the test was performed in triplicate. After the given time, the samples were transferred into sterilized centrifuge tubes containing 3 mL of LB medium. The tubes were shaken for 30 sec to remove the bacteria from the sample surface. Then, from the detached bacteria suspension 30 μL were evenly spread onto LB agar plates. These agar plates were placed in an incubator for 24 h at 37 °C in order to visualize the bacterial growth and / or inhibition. High resolution images of the agar plates were taken and further processed by using ImageJ 1.5i software to calculate the percentage of area covered by the bacterial colonies. For this purpose, images were converted to 8 bit, and a clear contrast was produced between the black background pixel and the white area as bacterial pixel by adjusting the threshold. The percentage of the area due to white pixels was considered a measure of the bacterial growth. 

#### 3.4.8. In Vitro Cell Culture Test

The in vitro cytocompatibility of the coatings was evaluated using the MG-63 osteoblasts cell line (Sigma-Aldrich, Taufkirchen, Germany). Initially, all coatings were sterilized under UV light for 1 h. A CS coating without copper was used as a control. Cells were cultured in cell culture polystyrene flasks using Dulbecco’s modified Eagle’s medium (DMEM, Gibco, Schwerte, Germany), supplemented with 10 vol. % fetal bovine serum (FBS, Sigma-Aldrich, Taufkirchen, Germany) and 1 vol. % penicillin/streptomycin (Pen-Strep; Sigma-Aldrich, Taufkirchen, Germany). When cell confluency reached up to 80%, a monolayer of the cells was detached from the flask’s wall using trypsin/EDTA solution (Life Technologies, Darmstadt, Germany) in PBS. After cell detachment, trypsination was inactivated by adding fresh DMEM, and the cell suspension was counted in a hemocytometer by trypan blue exclusion method (Sigma-Aldrich, Taufkirchen, Germany). A cell suspension of 10^5^ cells per mL was prepared, and 1 mL of this solution was used to directly cover the sterilized samples in 24-well plates. The cells were then allowed to grow on all coatings for 24 h at 5 % CO_2_ and 37 °C.

Water-soluble tetrazolium salt (WST-8 assay kit, Sigma-Aldrich, Taufkirchen, Germany) was used to evaluate the cell viability of MG-63 cells. Culture medium was removed completely from the wells after 24 h of incubation. Samples were washed with PBS and after that freshly prepared DMEM containing 1 vol. % WST-8 reagent was added in each well and incubated for 3 h. Subsequently, 100 μL from each well were transferred in a 96-well plate and the absorbance was measured at 450 nm with a micro plate reader. The cell viability (%) was calculated using Equation (5) from the optical density (OD sample) of each coating, of the WST reactant (OD blank) and of the CS coating (OD reference):

(5) $$\mathrm{Cell}\;\mathrm{viability}\;\left[\%\right]=\frac{OD_{\mathrm{sample}}-OD_{\mathrm{blank}}}{OD_{\mathrm{reference}}-OD_{\mathrm{blank}}}\times 100$$

The cell morphology and viability were qualitatively assessed by using live staining with DAPI (4′,6-diamidino-2-phenylindole) and Calcein AM (Life Technologies, Darmstadt, Germany). The staining was carried out by following the manuals provided by the supplier. Finally, images of Calcein-DAPI-stained samples were taken with a fluorescence microscope (Axio Scope A1, Carl Zeiss Microimaging GmbH, Jena, Germany).

### 3.5. Statistical Analysis

Statistical analysis was performed using one-way ANOVA test, with *p* < 0.05 (*) considered as being statistically significant. The experimental results are represented as mean values and standard deviations (SDs). The number of replicates ranged from three to six, depending on the test.

## 4. Conclusions

For the first time, we successfully synthesized Cu(II)–CS complexed coatings on a 316L stainless substrate using EPD. Physicochemical investigations confirmed that the obtained coatings were uniform and crack-free. Moreover Cu(II) ions produced successful chelated complex with CS, which was verified by FTIR and EDX results. Scratch and nano-indentation tests of all coatings showed improved mechanical properties up to a certain Cu concentration (%). EDX mapping confirmed the uniform distribution of Cu(II) on the surface of the coatings, which gave rise to superior antibacterial effects. In vitro cell studies confirmed the cytocompatibility of the coatings up to a certain concentration of Cu(II) ions. However, higher concentrations gave rise to cytotoxic effects to human osteoblast-like cells. Clearly, a more detailed study will be required to establish the relationship between coating composition and antimicrobial activity—for example, by carrying out XPS analysis of the films. Overall, the Cu(II)–CS complexed coating showed great potential for improving the biological performance of implants, although in vivo evaluations are essential for future developments toward clinical applications. 

## Figures and Tables

**Table 1:** Zeta potential and electrophoretic mobility of the suspensions used for electrophoretic deposition (EPD).

Sample	Zeta Potential (mV) at pH 4.7	Zeta Potential Dev. (mV)
**CS**	+29	6
**Cu(II)–CS1**	+29	6
**Cu(II)–CS2**	+21	6
**Cu(II)–CS3**	+22	6
**Cu(II)–CS4**	+22	7

**Table 2:** Calculated amount of Cu in the coatings by using energy-dispersive X-ray (EDX).

Samples	Cu(II)–CS1	Cu(II)–CS2	Cu(II)–CS3	Cu(II)–CS4
Theoretical X %	3	6	12	18
Cu_kL_/ C_kα_ %	2.93 ± 0.23	5.82 ± 0.28	11.45 ± 0.22	16.50 ± 0.27

**Table 3:** Critical load values in scratch test with respect to standard deviations of CS and its complex coatings.

Samples ID	CS	Cu(II)–CS1	Cu(II)–CS2	Cu(II)–CS3	Cu(II)–CS4
Critical load (N)	2.2	2.8	3.7	3.7	3.4
SD	0.1	0.1	0.2	0.3	0.2

**Table 4:** Amounts of Cu^2+^ in CS with different molar ratios and crossonding sample labelling.

Sample Labels	X (%)	Cu^2+^: NH_2_
**CS**	0	-
**CuCS1**	3	1:33
**CuCS2**	6	1:17
**CuCS3**	12	1:8
**CuCS4**	18	1:6
